# Plasma metabolome and skin proteins in Charcot-Marie-Tooth 1A patients

**DOI:** 10.1371/journal.pone.0178376

**Published:** 2017-06-02

**Authors:** Beatriz Soldevilla, Carmen Cuevas-Martín, Clara Ibáñez, Fulvio Santacatterina, María A. Alberti, Carolina Simó, Carlos Casasnovas, Celedonio Márquez-Infante, Teresa Sevilla, Samuel I. Pascual, María Sánchez-Aragó, Carmen Espinos, Francesc Palau, José M. Cuezva

**Affiliations:** 1 Departamento de Biología Molecular, Centro de Biología Molecular Severo Ochoa, Consejo Superior de Investigaciones Científicas-Universidad Autónoma de Madrid (CSIC-UAM), Madrid, Spain; 2 Centro de Investigación Biomédica en Red de Enfermedades Raras CIBERER-ISCIII, Madrid, Spain; 3 Instituto de Investigación Hospital 12 de Octubre, Universidad Autónoma de Madrid, Madrid, Spain; 4 Instituto de Investigación en Ciencias de la Alimentación, Consejo Superior de Investigaciones Científicas (CIAL-CSIC), Madrid, Spain; 5 Nutritional Genomics and Food GENYAL Platform, IMDEA Food Institute, Madrid, Spain; 6 Unidad Neuromuscular, IIS Hospital Universitario de Bellvitge, IDIBELL, l’Hospitalet de Llobegrat, Spain; 7 Servicio de Neurología y Neurofisiología, IIS Hospital Universitario Virgen del Rocío, Sevilla, Spain; 8 IIS Hospital Universitari i Politecnic La Fe, Departamento de Medicina, Universidad de Valencia, Valencia, Spain; 9 IIS Hospital Universitario La Paz, IDIPAZ, Madrid, Spain; 10 Centro de Investigación Príncipe Felipe, Valencia, Spain; 11 Institut de Recerca Sant Joan de Déu, Barcelona, Spain; 12 Division of Pediatrics, Facultat de Medicina i Ciències de la Salut, Universitat de Barcelona, Barcelona, Spain; Saint Louis University, UNITED STATES

## Abstract

**Objective:**

Charcot-Marie-Tooth 1A (CMT1A) disease is the most common inherited neuropathy that lacks of therapy and of molecular markers to assess disease severity. Herein, we have pursued the identification of potential biomarkers in plasma samples and skin biopsies that could define the phenotype of CMT1A patients at mild (Mi), moderate (Mo) and severe (Se) stages of disease as assessed by the CMT neuropathy score to contribute to the understanding of CMT pathophysiology and eventually inform of the severity of the disease.

**Methods:**

We have used: (i) a high-throughput untargeted metabolomic approach of plasma samples in a cohort of 42 CMT1A patients and 15 healthy controls (CRL) using ultrahigh liquid chromatography coupled to mass spectrometry and (ii) reverse phase protein microarrays to quantitate the expression of some proteins of energy metabolism and of the antioxidant response in skin biopsies of a cohort of 70 CMT1A patients and 13 healthy controls.

**Results:**

The metabolomic approach identified 194 metabolites with significant differences among the four groups (Mi, Mo, Se, CRL) of samples. A multivariate Linear Discriminant Analysis model using 12 metabolites afforded the correct classification of the samples. These metabolites indicate an increase in protein catabolism and the mobilization of membrane lipids involved in signaling inflammation with severity of CMT1A. A concurrent depletion of leucine, which is required for the biogenesis of the muscle, is also observed in the patients. Protein expression in skin biopsies indicates early loss of mitochondrial and antioxidant proteins in patients’ biopsies.

**Conclusion:**

The findings indicate that CMT1A disease is associated with a metabolic state resembling inflammation and sarcopenia suggesting that it might represent a potential target to prevent the nerve and muscle wasting phenotype in these patients. The observed changes in metabolites could be useful as potential biomarkers of CMT1A disease after appropriate validation in future longitudinal studies.

## Introduction

Charcot-Marie-Tooth (CMT) disease, also referred as hereditary motor and sensory neuropathy, is the most common group of inherited neuromuscular disorder with a prevalence of 1/2,500 [1]. Currently, mutations in more than seventy genes have been described as causes of CMT and the list of genes is ever-growing (Neuromuscular Disease Centre, http://neuromuscular.wustl.edu/index.html). Disease onset is usually in the first two decades and the symptoms include distal weakness and muscle atrophy, loss of proprioception and pinprick sensation [2]. Based on electrophysiological and nerve biopsy findings [2] two major CMT types can be distinguished dysmyelinating CMT (or CMT1) and axonal CMT (or CMT2). The most common form of CMT is type 1A (CMT1A), which is due to a 1.4 Mb duplication of chromosome 17 containing the *PMP22* gene [3, 4]. CMT1A constitutes approximately 50% of CMT cases in most series reported [5–7]. Because of this, the majority of therapeutic strategies have been designed for CMT1A patients. Ascorbic acid, progesterone antagonists or recombinant human neuregulin-1 have been investigated as therapies in rodent models [8–10]. However, and to date, the clinical trials using ascorbic acid in humans have not shown an effect on disease severity and progression [11, 12] and the need of biomarkers [13] for assessing CMT disease severity, progression and response to therapy are urgently required.

Biomarkers for assessing CMT patients include nerve conduction velocities, skin biopsies [14] and CMT neuropathy scores [15, 16]. Recently, a new magnetization transfer ratio MRI (magnetic resonance imaging) assay has been reported as biomarker of nerve pathology in CMT patients that correlates with disability [17] and electrical impedance myography has been suggested as a potential biomarker for CMT patients [18]. Moreover, the disturbance of intramuscular water distribution followed by fat accumulation, quantifiable by MRI pattern of the muscle is also a powerful biomarker of disease progression [19]. However, the current scenario shows that no molecular markers of the disease and effective therapies are available for CMT patients being the clinical scores the most used tools to assess the evolution of the disease.

Within the research project TREAT-CMT (http://www.treat-cmt.es/index.php/en), we have performed a study searching for skin and plasma molecular markers of CMT1A patients. We have developed a high-throughput untargeted metabolomic approach to identify potential disease biomarkers unknown so far, in a cohort of 57 plasma samples of CMT1A patients (n = 42) and healthy subjects (n = 15). Moreover, reverse phase protein microarrays (RPPmA) [20, 21] have been used to investigate the expression of proteins of energy metabolism in a cohort of 83 skin biopsies from CMT1A patients (n = 70) and healthy subjects (n = 13). The results provide molecular markers of the CMT1A phenotype that deepen in the pathophysiology of the disease.

## Materials and methods

### Patient’s evaluation

Seventy patients with CMT subtype 1A were prospectively evaluated by neurologists at the Neuromuscular Clinics of the following University Hospitals of Spain: Bellvitge, Barcelona (n = 37), Virgen del Rocío, Sevilla (n = 18), La Fe, Valencia (n = 13) and La Paz, Madrid (n = 2). Evaluation consisted of a detailed history, a neurological examination and electrophysiological studies. Primary clinical outcome was the CMT neuropathy score (second version) (CMTNSv2) [16].

### Human samples

Skin biopsies from seventy patients with clinical and genetic diagnosis of CMT1A were studied. Patients were classified attending to disease progression into mild (30 biopsies, Mi), moderate (25 biopsies, Mo) and severely (15 biopsies, Se) affected groups by neurological and clinical examination through CMT neuropathy score (Mild: 0–10; Moderate: 11–20 and Severe: > 20) at the hospitals involved in the project. Moreover, skin biopsies of thirteen healthy subjects, approximately matched by age with the moderate CMT1A group were also collected and studied. Blood samples from 42 of the CMT1A patients (15 Mild; 18 Moderate and 9 Severe) and from 15 healthy subjects were also collected. Skin biopsies and plasma samples were obtained in consenting control and CMT1A patients and immediately frozen and stored at -80°C until processing. The protocol was approved by the Ethics Committee/Institutional Review Board of the Hospital Universitari Bellvitge, Barcelona (PR184/12), Hospital Universitario Virgen del Rocío, Sevilla (CEI 2012PI/164), Hospital Universitario La Fe, Valencia (CEIB-2011/0537), Hospital Universitario La Paz, Madrid (HULP-PI-1378) and Universidad Autónoma de Madrid (CEI 52–961). Patients gave written informed consent before biopsy procedure and blood sampling following the Declaration of Helsinki. The samples were coded for anonymity to protect patient confidentiality.

### Metabolite extraction

A total of 57 plasma samples from forty two CMT1A patients suffering from mild (n = 15), moderate (n = 18) and severe (n = 9) disease and including 15 samples from healthy subjects were processed. Plasma samples were thawed on ice immediately prior to metabolic extraction. Metabolites were extracted from the samples (200 μL) by adding 1 mL of cold (-20°C) methanol. After vortex-mixing the mixture was incubated at -20°C for 12 h and centrifuged at 24,000 x g for 15 min at 4°C. The supernatant (900 μL) was collected and dried under vacuum. The vacuum-dried samples were dissolved in 180 μL of water and directly analyzed by UHPLC-MS. Quality control (QC) samples for each group of subjects (QCCrl, QCMi, QCMo and QCSe) were obtained by mixing equal volume of metabolic extracts from each group (i.e. control, mild, moderate and severe). Then a total QC (QCTotal) was also obtained by mixing equal volumes of the QC samples obtained for each of the groups (QCCrl, QCMi, QCMo and QCSe). These pooled samples were injected each 3 samples in order to monitor quality of analysis and to assist in data mining process. In addition, a mixture of commercial standards was injected each 5 runs in order to gain reliable data and to assure a correct instrument intra and inter-day variability. The commercial standard mixture consisted of 50 μM carboxymethylcysteine, 10 μM nicotinic acid, 8 μM adenosine, 80 μM benzoic acid, 100 μM N-benzoyltyrosine, 20 μM glutaric acid, 20 μM glucose-cysteine, 20 μM leucine, phenylalanine, 20 μM pantothenic acid and 20 μM methyl-2-aminobenzoic acid, in water. Only standards not detected in the plasma samples and not overlapping with the metabolic signal from the samples were evaluated. As a result a final concentration of 2 μM nicotinic acid and 100 μM N-benzoyltyrosine in the metabolic extracts was selected as internal standards.

### Metabolomic analysis

An untargeted or global metabolomic approach (https://www.ebi.ac.uk/training/online/course/introduction-metabolomics/designing-metabolomics-study/key-stages-metabolomics-study) was followed with the purpose of determining as many metabolites as possible without any bias. Metabolomic analyses were performed using a ultra-high performance liquid chromatography (UHPLC) system 1290 from Agilent (Agilent Technologies, Santa Clara, CA, USA) connected to a quadrupole-time-of-flight mass spectrometer (Q/TOF MS) Agilent 6540 equipped with an orthogonal ESI interface (Agilent Jet Stream, AJS) and operating in positive ion mode. The instrument was controlled by a PC running the Mass Hunter Workstation software 4.0 (MH) from Agilent. Reverse-phase (RP) chromatographic separation was performed using as phase A water with 0.1% formic acid (v/v) and as phase B acetonitrile with 0.1% formic acid (v/v). The final selected gradient was as follows: 0–30% B in 0–7 min, 30–100% B in 7–11 min, and 100% B in 11–14 min. The volume of sample injected was also optimized (2–4 μL) and 2 μL was the only volume of sample that did not saturate the MS detector. Each sample was analyzed in triplicate and three blanks were run between samples in order to avoid carryover of compounds between runs. MS operation parameters were the following: capillary voltage, -4000 V; nebulizer pressure, 30 psi; drying gas flow rate, 10 L/min; gas temperature, 300°C; skimmer voltage, 45 V; fragmentor voltage was 125 V in positive mode. TOF MS accurate mass spectra were recorded across the range of 50–1000 m/z at 1.5 spectra/s. Internal mass calibration of the instrument was carried out using an AJS ESI source with an automated calibration delivery system. The reference compound solution for internal mass calibration of the Q/TOF mass spectrometer containing 5 μM of purine ([C5H5N4]+ at 121.050873 m/z) and 2.5 μM HP-0921, hexakis (1H,1H,3H-tetrafluoropropoxy) phosphazine ([C18H19O6N3P3F24]+ at 922.009798 m/z) in acetonitrile-water (95:5, v/v) was also from Agilent. External calibration of the TOF MS was carried out using a commercial mixture from Agilent with next m/z values: 118.086255, 322.048121, 622.028960, 922.009798, 1221.990637 and 1521.971475.

### Data analysis and metabolite identification

Raw UHPLC-MS data were extracted and converted to the MS exchange format mzXML using the Trapper program version 4.3.0 (http://tools.proteomecenter.org/wiki/index.php?title=Software:trapper). Data processing was performed using R programming language (version 3.1.2) and Java. R package XCMS PeakML (http://masspec.scripps.edu/xcms/download.php) was used for peak detection. It converts three-dimensional LC-MS data (m/z, retention time and ion intensity) to a readily accessible two-dimensional data matrix as ion peaks (as pairs of their m/z and retention time) and their respective areas. Then to combine and align peaks from different samples mzMatch [22] was used. Gapfiller was used for recursive analysis (settings used for data processing in this work are described in S1 Table). Final filtering of the data was then carried out to ensure ions with a high quality: (i) peaks below 3x intensity from the blanks were removed, (ii) peaks not found in at least a 75% of the samples belonging to the same group of subjects (i.e. control, mild, moderate and severe groups of subjects) or with a high variability within the same group (with a value of median/average > 1.5) were removed. The resulting output data table of high quality time-aligned detected compounds, with their corresponding retention time, *m/z* and peak area obtained for each sample, was submitted to statistical analysis (see S2 Table).

Principal Component Analysis was applied to detect possible outliers in each group of samples (i.e. control, mild, moderate and severe groups). Once outliers were discarded a Kolmogorov-Smirnov test was applied to statistically evaluate normality by comparing the cumulative distribution of the data. Then Levene’s test was applied to assess the equality of the variances of the metabolites in all the groups. Analysis of variance by a parametric one-way ANOVA was performed to detect the different peak areas averages (p<0.05). A non-parametric analysis of variance applying a Kruskal-Wallis test was performed to compare the medians of the variables with heterogeneity in the variances among the four groups (revealed by Levene’s test). A Linear Discriminant Analysis (LDA), with forward stepwise procedure (with F values set at 4 and 3.9 to enter and to remove variables respectively) was performed to select the significant variables (p<0.05) most useful in differentiating the four sets of samples. Canonical Variate Analysis (CVA) was also carried out to obtain a low-dimensional graphical representation of the samples that separates the groups as much as possible. The predictive power of the LDA classification method was carried out by using a Leave-One-Out Cross-Validation procedure. Finally, we examined the differences in the average values corresponding to the LDA selected variables. STATISTICA (v.9, Statsoft, Tulsa, OK, USA, www.statsoft.com) and SPSS (v.19, IBM, Chicago, IL, USA, www.spss.com) programs for Windows were used for the statistical analysis. Tentative identification was carried out for statistically different (p<0.05) metabolites.

Tentative identification of key metabolites was achieved by matching the obtained accurate m/z to those published in appropriated databases: KEGG [23], HMDB [24] and Metlin [25] within a mass accuracy window of 10 ppm (see S2 Table). Molecular Formula Generator algorithm within MassHunter software (Agilent) was used to support the agreement between the molecular formula generated by the software and the proposed compound from metabolite database search in terms of mass error (ppm) and isotopic pattern similarity. When isomers existed for a given formula, metabolite identification was sorted giving preference to metabolites from central metabolic pathways in KEGG, metabolites already found in plasma and number of databases containing each metabolite.

When available, co-injection of standards with plasma samples was performed to confirm the identified metabolites. Leucine, lysine, tryptophan, sphingosine-1-phosphate, lyso-phosphatidylcholine (18:0), uridine and 2-deoxygalactopyranose, were from Sigma-Aldrich and urobilinogen was obtained from Santa Cruz Biotechnology (Santa Cruz, USA), and used for identification purposes.

### Preparation of skin extracts

Tissue powder obtained from frozen skin biopsies grinded in a mortar with liquid nitrogen were extracted with 150 μl of 50 mM Tris-HCl pH 8, containing 100 mM NaCl, 1 mM DTT, 1% (v/v) Triton X100, 0.1% SDS, 0.4 mM EDTA and a cocktail of protease (Roche) and phosphatase (Sigma) inhibitors. After protein extraction, samples were centrifuged (15,000 g) at 4°C for 30 min. The protein concentration in the supernatants was determined with the Bradford reagent (Bio-Rad Protein Assay) using BSA as standard. Aliquots of the supernatants were stored at -80°C until used.

### Printing and processing of reverse phase protein microarrays (RPPmA)

Protein samples from skin biopsies were diluted in PBS to a final protein concentration of 0.75 μg/μl before printing. Serially diluted protein extracts (0–1 μg/μl) derived from HCT116 and OVCAR8 cells were also prepared to asses printing quality and the linear response of protein recognition by the antibodies used. A solution of BSA and of mouse IgGs (1 μg/μl) were also prepared for printing as internal negative and positive control, respectively. Approximately, 1 nl volume of each sample was spotted in duplicate onto nitrocellulose-coated glass slides (FAST Slides, Scheleicher & Schuell BioScience, Inc.) using a BioOdyssey Calligrapher MiniArrayer printer (Bio-Rad Laboratories, Inc.) equipped with a solid pin (MCP310S) at constant humidity of 45% and 10°C and 16°C for the plate and chamber, respectively. Each array was incubated with each antibody independently. The commercial and/or in-house produced antibodies used in RPPmA were previously validated by western blotting of fractionated human tissue proteins in SDS-9% PAGE. The antibodies selected only recognized a single protein band of the expected molecular mass in different human tissues (See S1 Fig). A representative protein microarray illustrating the printing protocol of skin biopsies developed with antibodies against pyruvate kinase M2 (PKM2) is shown in S2 Fig.

After printing, arrays were allowed to dry at room temperature for 16 h and further blocked in PBS-T containing 5% skimmed milk. After, the arrays were incubated overnight at 4°C with the indicated concentrations of the following highly specific primary monoclonal antibodies (mAbs): anti-β-F1-ATPase (1/2,500), anti-PK-M2 (1/150), anti-GAPDH clone 273A-E5 (1:250) and anti-HSP60 clone 17/9-15G1 (1:250) from [26]; anti-IF1 (1/100) from [27]; anti-LDHA (1/2,500) from [21]; anti-HADHA (1/100), anti-COXII (1/100), anti-NDUFS3 (1/100) and anti-SOD2 from Abcam; anti-SDHB (1/500) and anti-PDHe (1/200) from Invitrogen; anti-Core 2 of complex III (1/1,000) from Mitosciences; anti-catalase (1/5,000) and anti-β-actin (1/5,000) from Sigma-Aldrich; anti-DLP-1 (1/200) from BD Transduction Lab and anti-G6PDH (1/500) from Thermo Scientific).

After incubation the arrays were washed with PBS-T and further incubated with a donkey anti-mouse or donkey anti-rabbit secondary antibody conjugated with alexa-488 (Invitrogen) and processed as previously described [20, 21]. Microarrays were scanned using a Typhoon 9410 scanner (GE Healthcare, Inc.). The mean fluorescent intensity of the spots was quantified using FIJI software (N.I.H., USA) and converted into arbitrary units of expressed protein/ng of total protein in the tissue extract using the expression obtained in the linear plot of HCT116 cell line protein.

### Other statistical analysis

Statistical analysis was performed by SPSS 23.00. Distribution of metabolic and protein markers and other categorical variables were contrasted using Kruskal-Wallis and t-test following evaluation of equality of variances or not with Levine´s test. Person and Spearman correlations analysis were used for the variables which follow normal and non-normal distribution respectively. Nonparametric receiver operating characteristic (ROC) curves were generated to plot sensitivity of some of the plasma metabolites against the false-positive rate (1-specificity). The relative ability of the biomarker in diagnosis was evaluated by calculating the area under the curve (AUC), and AUCs were compared by Chi-square test. P-values of less than 0.05 were considered statistically significant.

## Results

### Metabolic fingerprint in plasma of CMT1A patients

**Fig 1:**
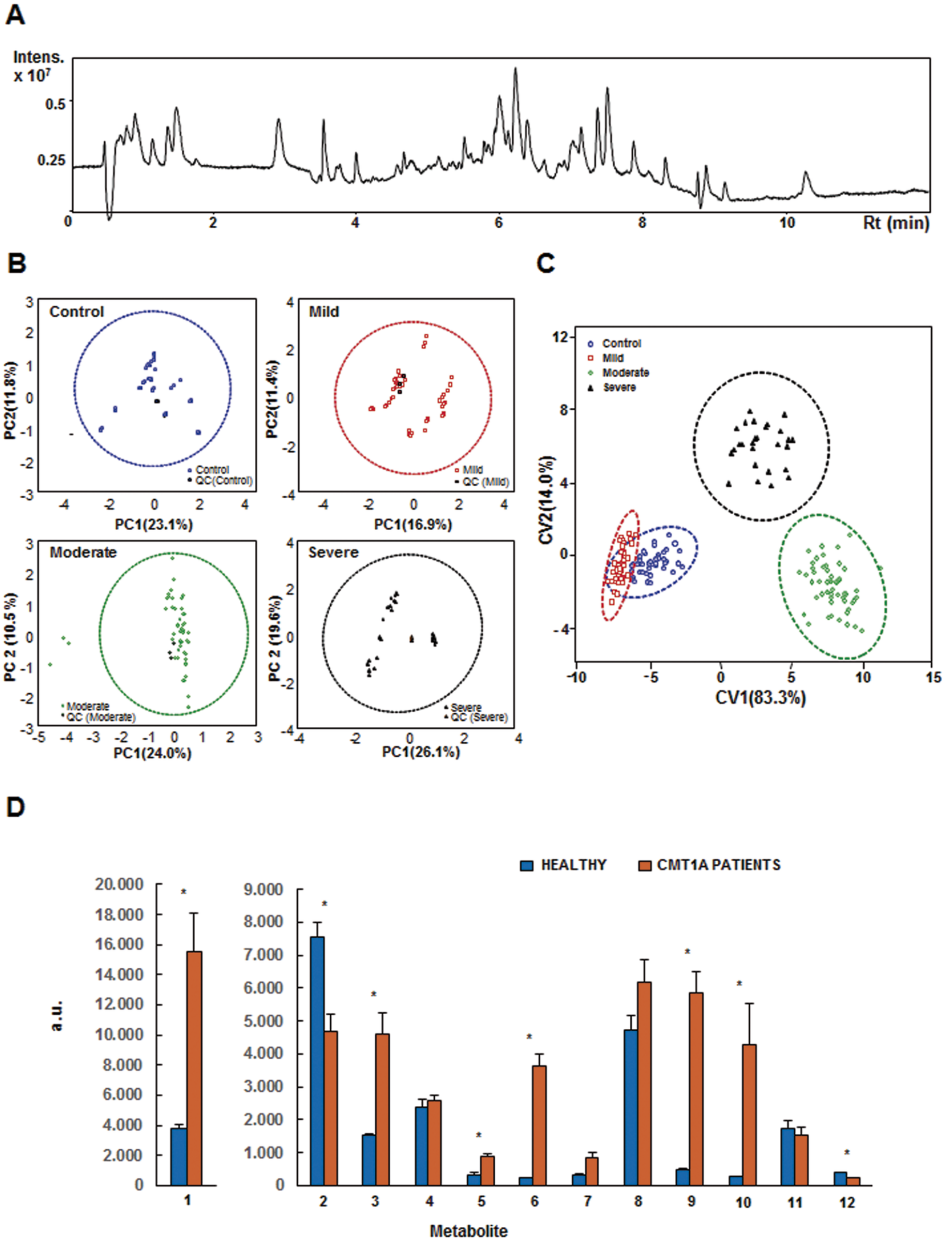
Analysis of plasma metabolome in CMT1A patients. (A), Representative UHPLC-MS total ion chromatogram of plasma samples. (B), Plot in a two-dimensional Cartesian coordinate system, with the axes (principal components, PC) representing the greatest variations in the data of Control (blue), Mild (red), Moderate (green) and Severe (black) states related to CMT1A. Three quality control (QC) injections per group are also represented in the plot for the four groups of individuals. 95% confidence ellipses are also included. Triplicate outliers of one of the samples in the Moderate group fall out of the ellipse. (C), Plot of distribution of the plasma samples defined by the two canonical variables (CV1 and CV2) obtained by Canonical Variate Analysis considering the 12 selected metabolites after forward stepwise Linear Discriminant Analysis. The 95% canonical ellipses are also included. Control subjects and mild, moderate and severe CMT1A patients are represented by blue circles, red squares, green diamonds and black triangles, respectively. (D), Histogram showing the content of the 12 metabolites in plasma samples of healthy (blue, n = 15) and CMT1A patients (yellow, n = 42). The results shown are the mean values ± S.E.M. *, P<*0*.*05* by *Student’s t test*.

A representative UHPLC-MS metabolic profile of plasma samples is shown in Fig 1A. After data processing a total of 3,326 highly confident metabolite signals detected in all the samples were considered for analysis and the resulting data matrix was normalized and subjected to statistical analysis. After Principal Component Analysis, one sample in the moderate CMT1A group that fell out of the 95% confidence ellipse (Fig 1B) was considered outlier and removed from the statistical analysis. It should be noted that quality control samples (QC) are closely clustered in the middle of the score plots (Fig 1B), confirming the reliability and quality of the UHPLC-MS data (Fig 1A). Both parametric and non-parametric methods of univariate analysis showed 194 metabolite signals with significant differences among the groups of samples (see S2 Table). To select the signals most useful in discriminating the four groups a multivariate Linear Discriminant Analysis was applied revealing 12 metabolites able to correctly classify all samples. A Canonical Variate Analysis was carried out to obtain a low-dimensional graphical representation of the results (Fig 1C). The resulting plot showed a clear independent clustering of the four groups of samples (Fig 1C) being the metabolic fingerprints of CMT1A mild patients and healthy controls those more similar among the four groups of individuals studied (Fig 1C). The abundance of the 12 metabolites used in the multivariate Linear Discriminant Analysis model shows that 6 increase whereas 2 decrease in CMT1A patients when compared to healthy controls (Fig 1D).

**Fig 2:**
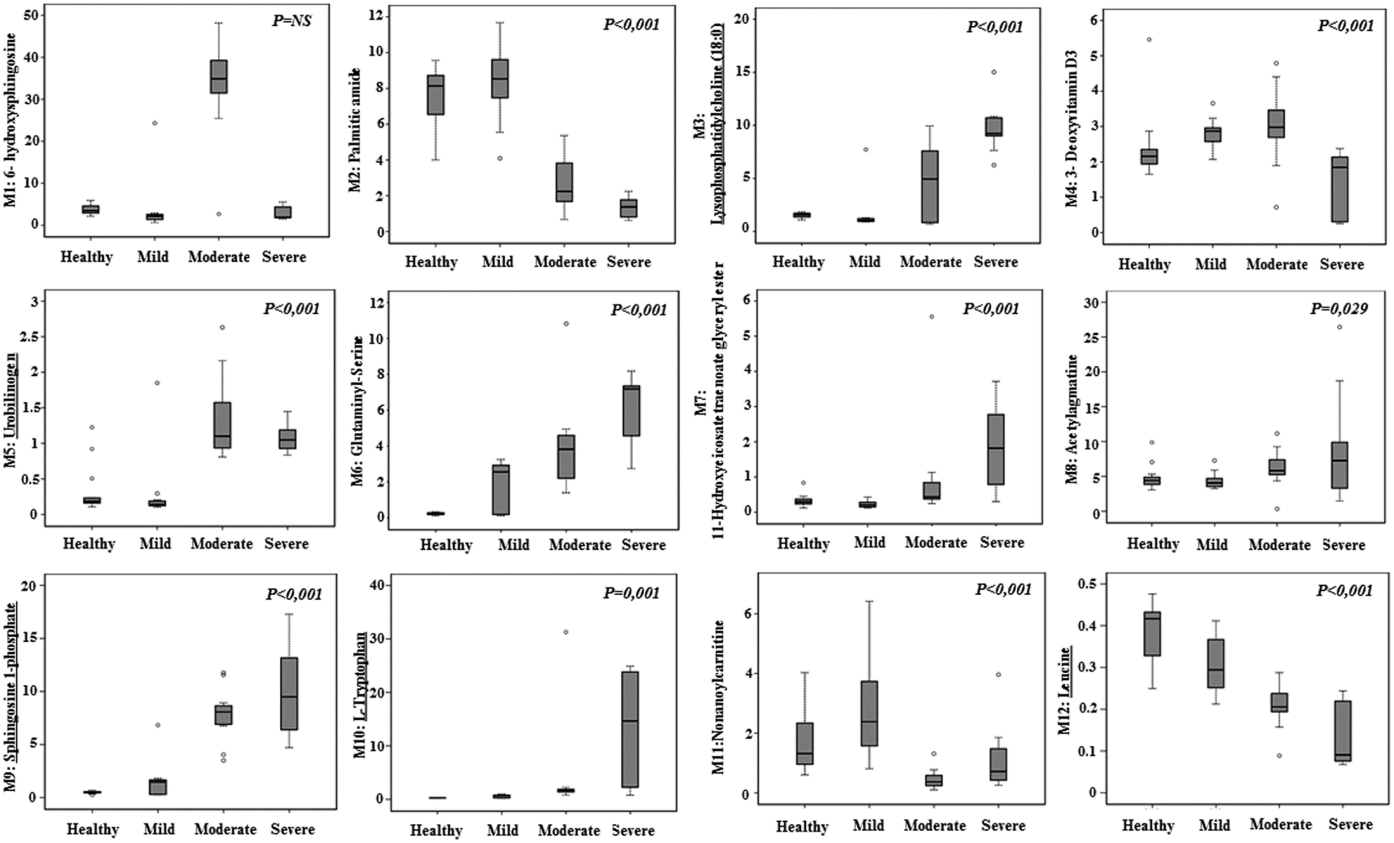
Whisker plots showing significant correlations between plasma metabolite levels and severity of the disease in CMT1A patients. Determination of the plasma levels of the 12 selected metabolites after forward stepwise Linear Discriminant Analysis was carried out by UHPLC-MS and grouped considering severity of the disease as assessed by the CMT neuropathy score (second version). Healthy subjects (n = 15), Mild (n = 15), Moderate (n = 18) and Severe (n = 9) groups of CMT1A patients are represented. Identification of the metabolites was achieved by matching the obtained accurate m/z to those published in appropriated databases (see S2 Table) and when available, by co-elution of commercial standards with the extracted ion chromatograms of plasma samples (highlighted in red). Box plots represent the lowest, lower quartile, median, upper quartile, and highest observations of each marker in the different groups. ○, outlier values P-*value* is calculated by analysis of variance; *r* is the *Spearman coefficient*.

As expected, the age and CMTNSvs2 of the CMT1A patients studied in the cohort used for metabolomic studies increased with progression of the disease (S3 Table). The differences in plasma values of the 12 metabolic signals selected for Linear Discriminant Analysis of the four groups of samples are summarized in Fig 2. There are two main trends of variation of the plasma metabolites: those that increase in content with severity of the disease, such as metabolites 3, 5, 6, 7, 8, 9, and 10; and those whose signals drop with disease severity such as metabolites 2, 4, 11 and 12 (Fig 2). Metabolite 1 escapes this classification (Fig 2).

**Fig 3:**
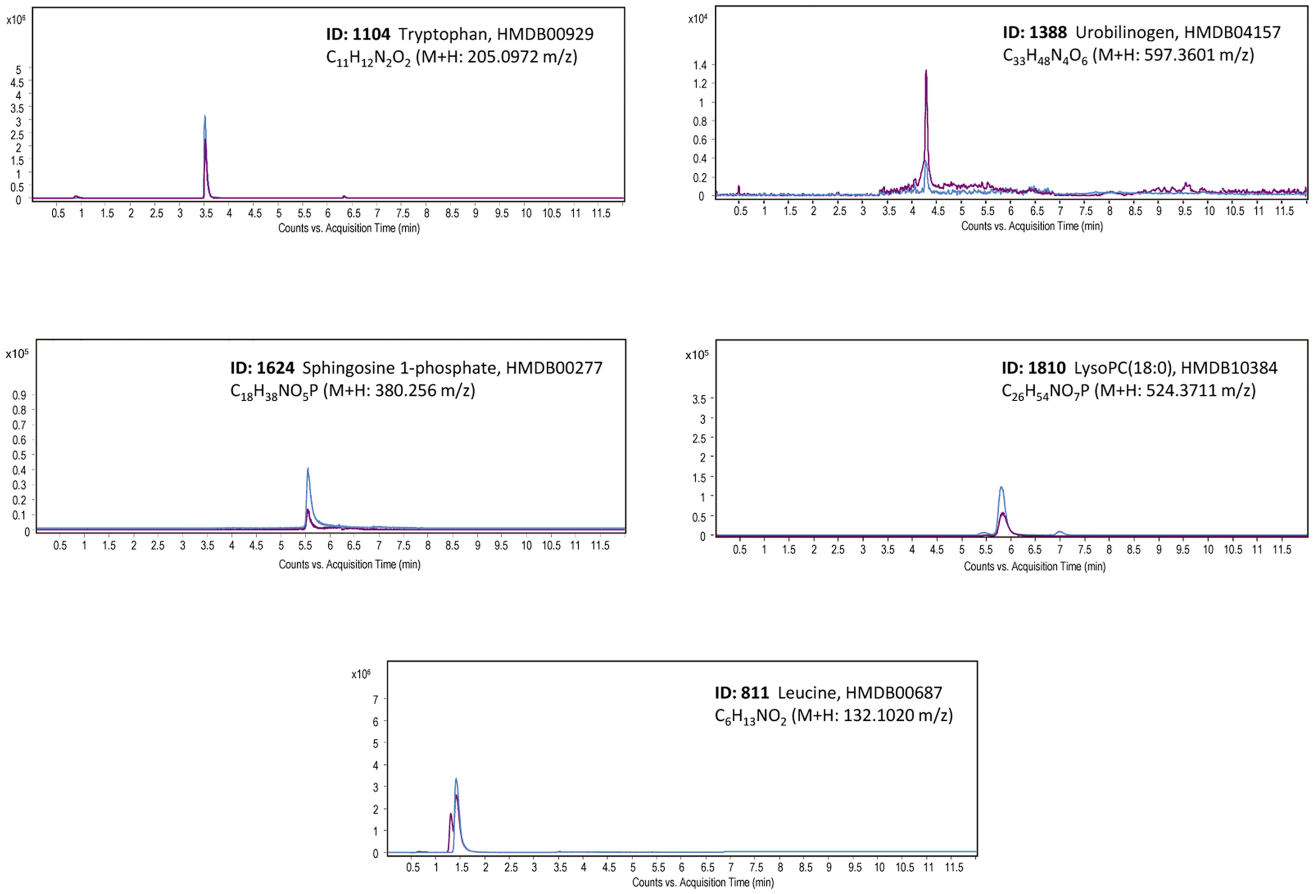
Identification of metabolites by co-elution with commercial standards. Extracted ion chromatograms of selected metabolites in plasma samples (purple lines) and commercial standards (blue lines) are shown. The metabolites are defined by their name, ID number, m/z (M+H), retention time, Human Metabolome Data Base ID number and molecular formula.

The identification of the 12 metabolites (Fig 2) was carried out as described in Methods (see S2 Table for details) and leucine, tryptophan, sphingosine-1-phosphate, lyso-phosphatidylcholine (18:0) and urobilinogen confirmed as potential biomarkers of CMT1A disease by co-injection of the commercially available standards (Fig 3, and see S2 Table for details). Moreover, lysine, uridine and 2-deoxygalactopyranose (see S2 Table) were also confirmed by co-injection with appropriate standards (S3 Fig). Severity of CMT disease correlates with an increase in the age of patients (S3 Table). In this regard, it should be noted that of the twelve metabolites used in the LDA (Fig 2) only 3-deoxyvitamin D3 significantly correlated with age of the patients; a finding that was confirmed by calculation of the Partial Correlation Coefficient (PCC) that measures the association between the three variables.

**Fig 4:**
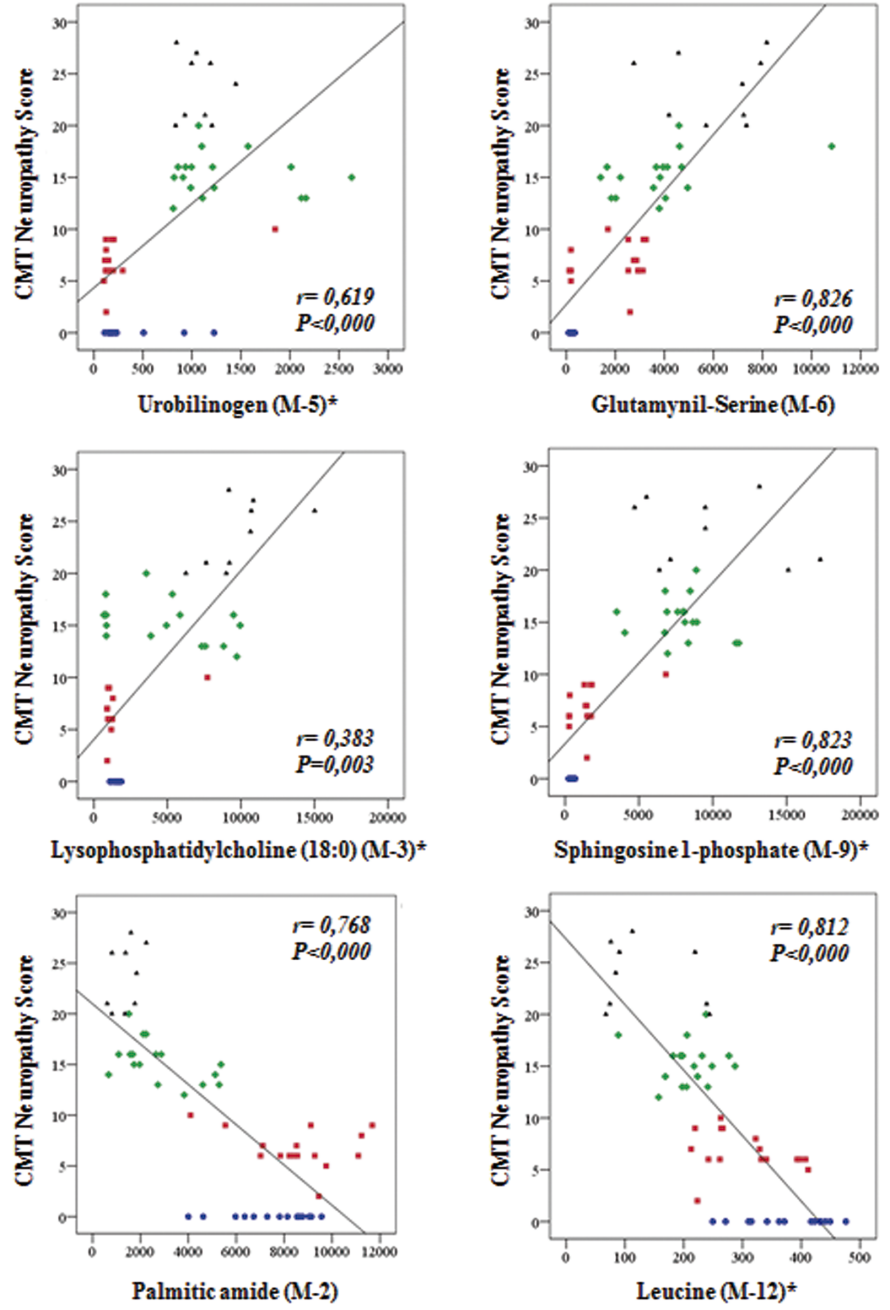
Scatter plots showing significant correlations between plasma metabolite levels and severity of the disease in CMT1A patients. Determination of the plasma levels of the metabolites was carried out by UHPLC-MS and its levels correlated with severity of the disease as assessed by CMTNSv2. Healthy controls (blue circles, n = 15), Mild (red squares, n = 15), Moderate (green diamonds, n = 18) and Severe (black triangles, n = 9) groups of CMT1A patients are represented. Metabolites related to (i) protein catabolism (upper row); (ii) mobilization of membrane lipids (middle row) and (iii) muscle biogenesis and the anti-apoptotic function (lower row) are represented. P-value is calculated by analysis of variance; r is the Spearman coefficient.

**Fig 5:**
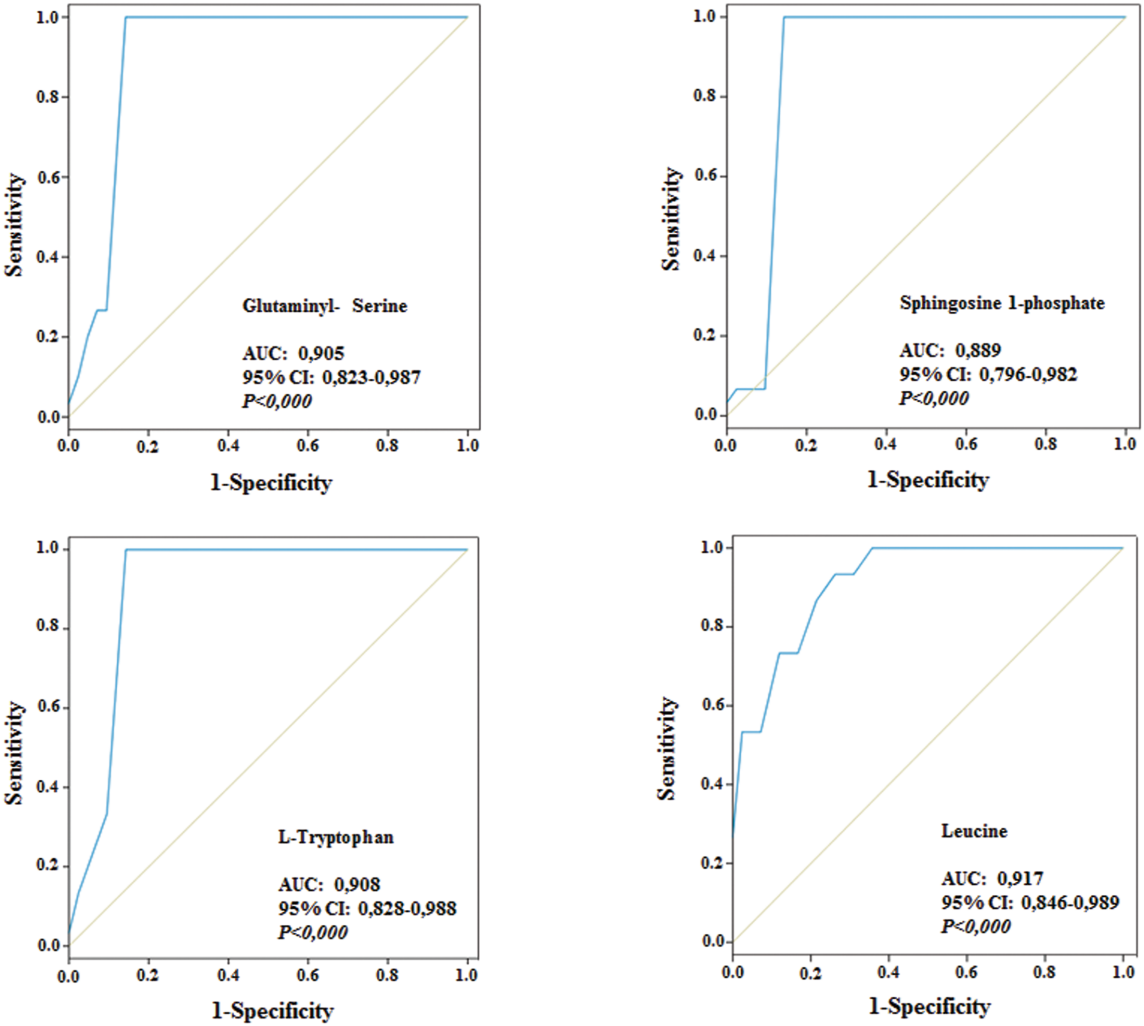
Diagnostic performance of metabolic biomarkers. ROC curves were plotted to describe performance characteristics of the indicated metabolites in the 57 subject cohort. The Area under the curve (AUC), 95% range of the interval of confidence (IC) and P values are indicated.

Interestingly, and with the exception of 6-hydroxysphingosine, the identified metabolites could be classified into two major groups attending to its trend of variation with severity of the disease as assessed with the CMTNSvs2 (Fig 2). Those that increase in the severe group correspond to metabolites revealing an enhanced protein catabolism such as urobilinogen and glutaminyl-serine (Fig 4) and the mobilization of membrane lipids involved in inflammation, immune response and quimiotaxis (lysophosphatidylcholine and sphingosine-1-phosphate (Fig 4). Those that decrease with severity of the disease correspond to metabolites such as leucine that is essential for the biogenesis of the muscle and of palmitic amide, a poorly characterized disease marker that might play an anti-apoptotic function (Fig 4). Interestingly, of the 12 metabolites used to develop the LDA, four of them (glutaminyl-serine, sphingosine-1-phosphate, tryptophan and leucine) could provide potential biomarkers of the disease as assessed by their significance in ROC curves (Fig 5), but after approval in longitudinal, interpersonal and interinstitutional studies [13].

### Protein fingerprint of CMT1A skin biopsies

S3 Table summarizes the progressive increase in age and CMTNSvs2 of the cohort of CMT1A patients studied. The analysis of protein expression in skin biopsies comparing all data from CMT1A patients to the controls provided significant differences in mitochondrial proteins of the OXPHOS system (Table 1) which included representative markers of the electron transport chain (NDUFS3 of Complex I, SDH-B of Complex II, Core 2 of Complex III and COXII of Complex IV) and oxidative phosphorylation (β-F1-ATPase and IF1). Other mitochondrial proteins involved in mitochondrial structure (Hsp60) and dynamics (DLP-1), pyruvate metabolism (PDHe) and β-oxidation (HADHA) and the antioxidant response (SOD2) were not significantly affected (Table 1). Likewise, the expression of proteins of glycolysis (GAPDH, PK-M2 and LDHA) and of the pentose phosphate pathway (G6PDH) was not affected (Table 1). Interestingly, the expression of catalase was significantly reduced in skin biopsies of CMT1A patients (Table 1). Depletion of mitochondrial markers in skin biopsies occurred early during onset of the disease because for most of the CMT1A down-regulated proteins we observed that its expression in early stage patients was not different from that at later stages of the disease (Table 1). It should be noted that some of the down-regulated proteins showed an enhanced expression at later stages of disease (Table 1). However, Spearman test indicated that none of the protein markers correlate with disease severity as assessed by CMTNSv2. It should be noted that peripheral nerves represent a small fraction of skin biopsies. Therefore, the changes reported in protein expression might be biased toward the abundant skin proteins missing other relevant nerve proteins for pathogenesis.

**Table 1:** Protein expression in skin biopsies of CMT1A patients.

		CMT1A				
Protein Markers		Healthy	TOTAL	MILD	MODERATE	SEVERE
**N° cases**		13	70	30	25	15
**Glycolysis**	**GAPDH**	0.98 ± 0.13	0.84 ± 0.04	0.95 ± 0.08	0.73 ± 0.07	0.79 ± 0.09
	**PKM2**	0.87 ± 0.13	1.00 ± 0.09	1.29 ± 0.18	0.78 ± 0.07	0.79 ± 0.12
	**LDHA**	1.23 ± 0.14	1.08 ± 0.05	1.15 ± 0.10	1.04 ± 0.08	0.98 ± 0.08
**OXPHOS**	**NDUFS3**	5.47 ± 1.39	3.46 ± 0.27*	3.29 ± 0.43	3.48 ± 0.41	3.78 ± 0.70
	**SDHB**	3.82 ± 0.86	2.65 ± 0.17*	2.63 ± 0.25	2.70 ± 0.32	2.61 ± 0.38
	**CORE2**	1.50 ± 0.22	1.14 ± 0.06*	1.21 ± 0.11	1.11 ± 0.09	1.03 ± 0.15
	**COXII**	1.78 ± 0.32	1.26 ± 0.07*	1.29 ± 0.10	1.28 ± 0.13	1.16 ± 0.16
	**βF1**	1.19 ± 0.11	0.91 ± 0.05*	0.96 ± 0.09	0.87 ± 0.08	0.87 ± 0.11
	**IF1**	1.96 ± 0.51	1.23 ± 0.11*	1.06 ± 0.17^#^	1.38 ± 0.21	1.33 ± 0.20
**Mit. Structure, Dynamics and Metabolism**	**HSP60**	1.30 ± 0.27	0.87 ± 0.08	0.78 ± 0.05^#^	0.95 ± 0.20	0.91 ± 0.13
	**PDHe**	1.97 ± 0.43	1.56 ± 0.09	1.41 ± 0.13	1.63 ± 0.16	1.74 ± 0.24
	**HADHA**	1.56 ± 0.23	1.38 ± 0.10	1.34 ± 0.18	1.31 ± 0.13	1.55 ± 0.19
	**DLP1**	4.82 ± 1.25	3.32 ± 0.33	2.60 ± 0.49^#^	3.67 ± 0.46	4.17 ± 0.85^¥^
**Antioxidant System**	**SOD2**	12.70 ± 4.07	8.63 ± 0.84	6.40 ± 1.09^#^	8.56 ± 0.46	13.2 ± 2.45^¥^
	**CATALASE**	4.96 ± 0.97	3.19 ± 0.25*	2.59 ± 0.32^#^	3.48 ± 0.48	3.83 ± 0.50^¥^
**Pentose Phosphate Pathway**	**G6PDH**	3.78 ± 0.74	2.63 ± 0.25	1.91 ± 0.24^#^	2.81 ± 0.44	3.79 ± 0.70^¥^

The relative expression (a.u./ng protein) of relevant proteins involved in glycolysis (GAPDH, PKM2, LDHA), oxidative phosphorylation (OXPHOS, NDUFS3, SDHB, CORE2, COXII, βF1 and IF1), mitochondrial structure (HSP60), dynamics (DLP1) and metabolism (PDHe, HADHA) and of the antioxidant response (SOD2, catalase, G6PDH) as determined by reverse phase protein microarrays is shown for the cohort of biopsies studied and grouped in Healthy and CMT1A patients (total CMT1A cohort and for each of the mild, moderate and severe groups) as assessed by the CMTNSv2. The results shown are the mean values ± S.E.M.

*, ^#^ and ^¥^, *P<0*.*05* when compared healthy vs CMT1A Total, healthy vs Mild and Mild vs Severe, respectively. The *P-value* is determined by *t-test*.

## Discussion

The identification of biomarkers to aid the diagnosis and prognosis and eventually predict the response to treatment is a bottleneck for the innovation in medicine. In the present study, we utilized a large cohort of CMT1A patients to identify changes in skin proteins and plasma metabolites that may contribute to the understanding of the molecular basis of the disease and its severity and could eventually become future biomarkers of the disease [13].

Skin biopsies provide an important tool to the neurologist for the diagnosis of peripheral nerve disorders [14]. Herein, we have determined the relative expression of proteins involved in different metabolic pathways (glycolysis, oxidative phosphorylation, mitochondrial metabolism, structure and dynamics and of the antioxidant system) of skin biopsies of CMT1A patients, to understand the etiology of the disease and to be added as quantitative molecular markers to the changes in morphology and density of epidermal nerves and of other immunohistochemical studies that inform about the pathogenesis and prognosis of this peripheral neuropathy. If the reduction of epidermal nerve density is a common neuropathic abnormality in skin biopsies of CMT patients [14] we now show that the early loss of the mitochondrial enzymatic machinery involved in the provision of metabolic energy by oxidative phosphorylation and of ROS handling is also a distinctive feature of skin biopsies of CMT1A patients at early onset of the disease. This finding is in agreement with a similar observation in peripheral nerves of the *Gadp1* ^-^/^-^ mouse model of CMT disease [28]. Overall, the findings in human biopsies and in the *Gadp1* ^-^/^-^ mice further suggest that the bioenergetic deficit originated by mitochondrial dysfunction is likely to contribute to the loss of peripheral nerves in CMT1A disease.

High-throughput metabolomic technologies of plasma metabolites provide accessible and convenient biomarkers for diagnosis, prognosis and therapeutic intervention in a large number of human diseases [29, 30]. Herein, we have used liquid chromatography/mass spectrometry to detect 3,326 highly confident metabolite signals in the serum of healthy and CMT1A patients and identify 194 metabolites that reveal significant differences among the groups studied to examine their relation to CMT1A disease. We find that twelve metabolites allowed the discrimination of healthy subjects from affected patients and that the trend of variation and abundance of these metabolites nicely discriminate mildly, moderately and severely affected patients, emphasizing their potential as plasma metabolites that could aid the diagnosis and prognosis of CMT1A patients in future studies. Notably, the identified metabolites are either up-regulated or down-regulated with severity of the disease. Within the first group of up-regulated ones we find markers of protein catabolism such as the dipeptide glutaminyl-serine, tryptophan, urobilinogen (a marker of the degradation of heme containing proteins) and the polyamine acetylagmatine, all consistent with an enhanced breakdown of proteins that may be associated with peripheral nerve and muscle pathology in the patients. The polyamine, which results from the degradation of arginine, can also participate in CMT1A neuropathy since it has been reported to inhibit muscle contraction after electrical stimulation [31].

The other group of up-regulated metabolites is comprised by four lipid components released from biological membranes that are sphingosine-1-phosphate, 6-hydroxysphingosine, lysophosphatidylcholine and 11-hydroxyeicosatetraenoate glyceryl ester. The ceramide sphingosine-1-phosphate and lysophosphatidylcholine are signaling molecules recognized by specific plasma membrane G-protein-coupled receptors of the lysophospholipid receptor family [32]. Sphingosine-1-phosphate signaling is needed for egression of immune T and B cells from lymphoid organs into the blood and their circulation to inflamed tissues [33]. Lysophosphatidylcholine stimulates phagocyte recruitment and phagocytosis of the myelin sheath causing demyelination and mimicking the effects of multiple sclerosis [34, 35]. Like in the case of the ceramide, there are studies supporting that manipulating the inflammatory response provides a benefit in myelin repair in multiple sclerosis [34, 36–38]

The down-regulation of plasma leucine levels in the disease is difficult to rationalize based on a restriction of its ingestion in the diet of the patients. Leucine, is involved in the induction of muscle biogenesis [39] and is an independent predictor of muscle protein synthesis [40, 41]. Moreover, and since its plasma concentration does not correlate with the age of the patients, its downregulation is likely to result from either a diminished unexplained malabsorption of the essential amino acid and/or because its oxidation is enhanced with severity of the disease. The oxidation of the essential branched chain amino acid is indicative of a prolonged fasting and/or endurance exercise [42, 43]. This metabolic phenotype is associated with an enhanced plasma availability of fatty acids, products of the catabolism of triglycerides and membrane phospholipids, which favor the plasma accumulation of tryptophan [43–46]. The fate of the fatty acids liberated by the hydrolysis of membrane phospholipids is its oxidation in mitochondrial β-oxidation, which is also the final destiny of the carbon skeletons resulting from the oxidation of branched chain amino acids. Hence, the enhanced plasma availability of lysophosphatidylcholine and tryptophan and the reduction of plasma leucine support the idea of a prolonged and unexplained fasting-like metabolic state in severely affected CMT1A patients. Remarkably, the PROVIDE Study, a recent randomized, double-blind, placebo controlled trial has stressed the benefit of leucine plus vitamin D3 supplementation on measures of sarcopenia in older adults [47].

## Conclusion

**Fig 6:**
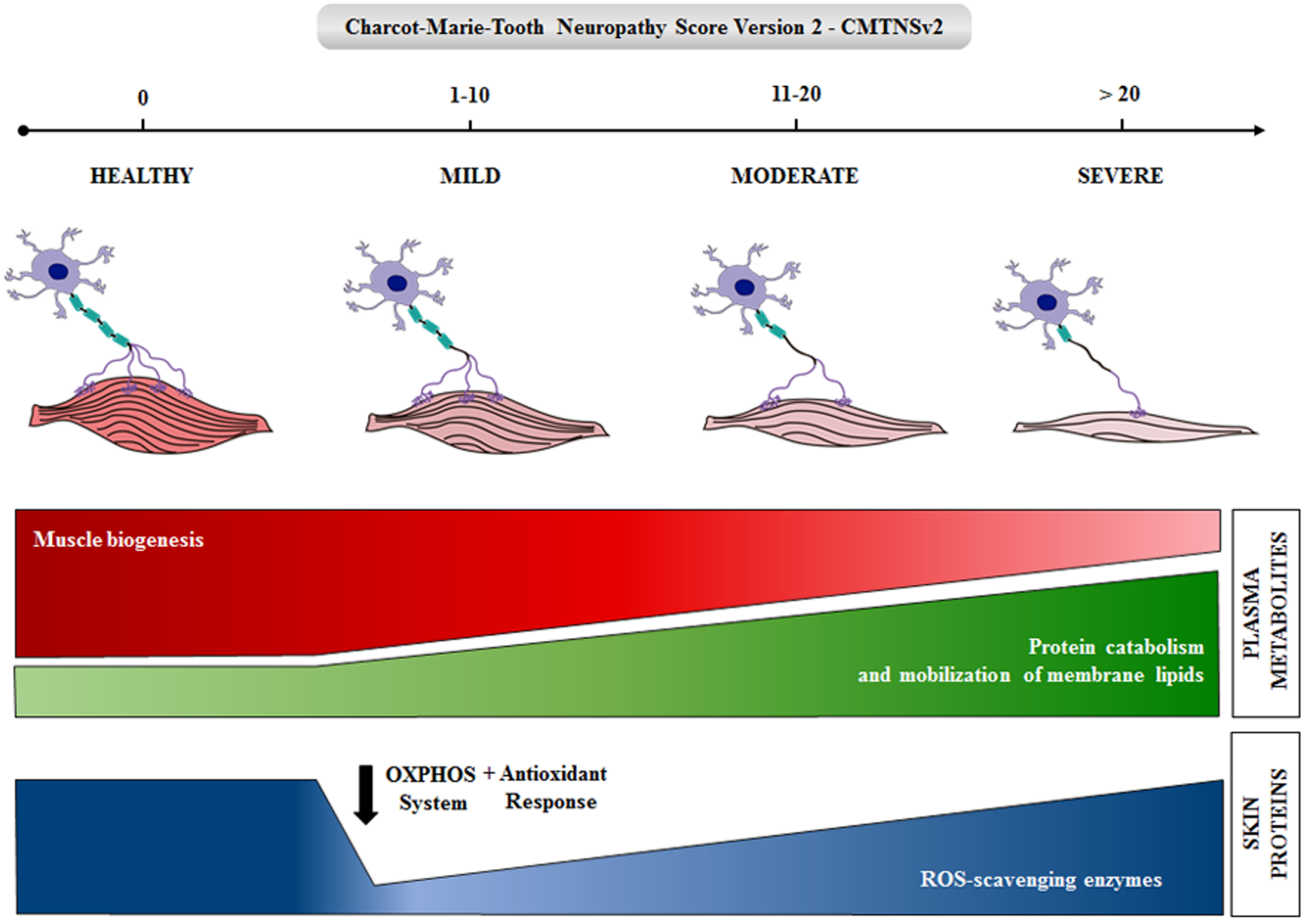
Summary of the major changes in the expression of skin and plasma metabolites during progression of CMT1A. The loss of muscle and nerves is shown during severity of the disease as revealed by the CMT neuropathy score v2. The changes in biomarkers are illustrated by row thicknesses.

We report significant changes in skin proteins and plasma metabolites that represent molecular candidates that might become biomarkers of CMT1A disease after appropriate future evaluation. The results indicate that an early event of the disease is the loss of mitochondrial bioenergetic markers in skin biopsies (Fig 6). Moreover, severity of the disease seems to be associated with a metabolic state resembling inflammation and sarcopenia (Fig 6), what could partially explain the nerve and muscle wasting phenotype in these patients. Some of the markers identified might contribute to monitor the effect of potential therapies in future clinical trials.

## Supporting information

S1 Table Parameters used for data processing. A total of 57,647 signals were detected and aligned in the plasma samples through data processing by means of R program. The subsequent peak filtering process allowed us to detect 3,326 high confident metabolites (plus the signals corresponding to the 2 internal standards) in all the samples (i.e. 171 replicates from CMT1A plasma samples and 12 selected replicates from QC samples).
(DOCX)

S2 Table Spreadsheet of output data. Lists the metabolite signals differentially expressed among the four groups of samples. All signals are defined by their retention time and a mass-to-charge (m/z) value. In addition, the 12 metabolites used in the LDA are highlighted in red.
(XLSX)

S3 Table Clinicopathological parameters of CMT1A patients in metabolomic and proteomic approaches. Number of cases, age and CMT neuropathy score (second version) of each cohort of patients is indicated for the Total CMT1A cohort and for each mild, moderate and severe groups studied. The results shown are the mean values ± S.E.M. The *P-value* is indicated as assessed by *Kruskal-Wallis test*.
(DOCX)

S1 Fig Validation of the antibodies used for application in RPPmA. 20–25 μg of protein derived from human muscle were fractionated on SDS-PAGE gels, blotted against the indicated antibodies and processed for western blotting. The migration of molecular mass markers is indicated to the left.
(PPTX)

S2 Fig Representative reverse phase protein microarrays showing the printing scheme of skin biopsies. **A,** Scheme of RPPmA printing processed for anti-PK-M2 is shown magnified. One nl samples were spotted in duplicate. Black boxed: negative controls of BSA; Blue and purple boxed: standard curves of HCT116 and OVCAR8 cells; Light blue: mouse IgGs; Green boxed: tissue samples from control donors; Red boxed: tissue samples from patients. Below are shown representative RPPmAs processed with other antibodies. **B,** The plot illustrates the linear correlation that exists between the fluorescence intensity (arbitrary units, a.u.) and the amount of PK-M2 in HCT116 cell lysates. Protein concentrations in the biopsies were calculated according to the fluorescence intensity obtained in the linear plot of HCT116 cells.
(PPTX)

S3 Fig Identification of metabolites by co-elution with commercial standards. Extracted ion chromatograms of selected metabolites in plasma samples (purple lines) and commercial standards (blue lines) are shown. The metabolites are defined by their name, ID number, m/z (M+H), retention time, Human Metabolome Data Base ID number and molecular formula.
(PPTX)

## Abbreviations

CMT: Charcot-Marie-Tooth
CMTNSvs2: CMT neuropathy score (second version)
RPPmA: Reverse phase protein microarrays
