# Supplementary material for: Plasma metabolome and skin proteins in Charcot-Marie-Tooth 1A patients
Source: PLoS One. 2017 Jun 2;12(6):e0178376. doi: 10.1371/journal.pone.0178376 (PMC5456076; doi:10.1371/journal.pone.0178376)
Supplement: S1 Table — A total of 57,647 signals were detected and aligned in the plasma samples through data processing by means of R program. The subsequent peak filtering process allowed us to detect 3,326 high confident metabolites (plus the signals corresponding to the 2 internal standards) in all the samples (i.e. 171 replicates from CMT1A plasma samples and 12 selected replicates from QC samples). (DOCX) [file pone.0178376.s001.docx]

| **Peak detection**  *(XCMS)* | **Peak alignment and combination**  *(mzMatch)* | **Recursive analysis**  *(PeakML.Gapfiller)* |
| --- | --- | --- |
| Method: Centwave  Tolerance = 40 (ppm)  Peak width = 5-100 (s)  S/N ratio = 3  Prefilter = at least 3 peaks at minimum intensity of 300  Method 1 for integration  Min difference in m/z for peaks overlapping retention times = 0·008 m/z | Ret time window = 30 (s) and 60 (s) for samples from the same group and to combine different groups of samples, respectively.  Tolerance = 20 (ppm) | Tolerance = 40 (ppm)  Ret time window = 30 (s) |
