# Supplementary material for: Plasma metabolome and skin proteins in Charcot-Marie-Tooth 1A patients
Source: PLoS One. 2017 Jun 2;12(6):e0178376. doi: 10.1371/journal.pone.0178376 (PMC5456076; doi:10.1371/journal.pone.0178376)
Supplement: S3 Table — Number of cases, age and CMT neuropathy score (second version) of each cohort of patients is indicated for the Total CMT1A cohort and for each mild, moderate and severe groups studied. The results shown are the mean values ± S.E.M. The P-value is indicated as assessed by Kruskal-Wallis test. (DOCX) [file pone.0178376.s003.docx]

|  |  |  | **CMT1A** | | | |  |
| --- | --- | --- | --- | --- | --- | --- | --- |
| **Cohort** | **Clinicopathological Parameters** | **Healthy** | **TOTAL** | **MILD** | **MODERATE** | **SEVERE** | ***P-value*** |
| **Metabolomic**  **Approach** | **Nº Cases** | 15 | 42 | 15 | 18 | 9 |  |
|  | **Age** | 42.07 ± 3.32 | 42.64 ± 2.62 | 35.15± 4.42 | 41.56 ± 3.52 | 57.33 ± 3.98 | *0.021* |
|  | **CMTNSv2** | - | 13.83 ± 0.16 | 6.53 ± 0.46 | 15.00 ± 0.56 | 23.67 ± 1.06 | *<0.001* |
| **Proteomic Approach** | **Nº Cases** | 13 | 70 | 30 | 25 | 15 |  |
|  | **Age** | 43.95 ± 3.38 | 44.45 ± 0.22 | 40.72 ± 2.59 | 43.47 ± 2.79 | 54.25 ± 2.53 | *0.026* |
|  | **CMTNSv2** | - | 12.23 ± 0.81 | 6.11 ± 0.51 | 14.73 ± 0.47 | 22.94 ± 0.70 | *<0.001* |
