# Supplementary material for: Plasma metabolome and skin proteins in Charcot-Marie-Tooth 1A patients
Source: PLoS One. 2017 Jun 2;12(6):e0178376. doi: 10.1371/journal.pone.0178376 (PMC5456076; doi:10.1371/journal.pone.0178376)
Supplement: S1 Fig — 20–25 μg of protein derived from human muscle were fractionated on SDS-PAGE gels, blotted against the indicated antibodies and processed for western blotting. The migration of molecular mass markers is indicated to the left. (PPTX) [file pone.0178376.s004.pptx]

## Slide 1
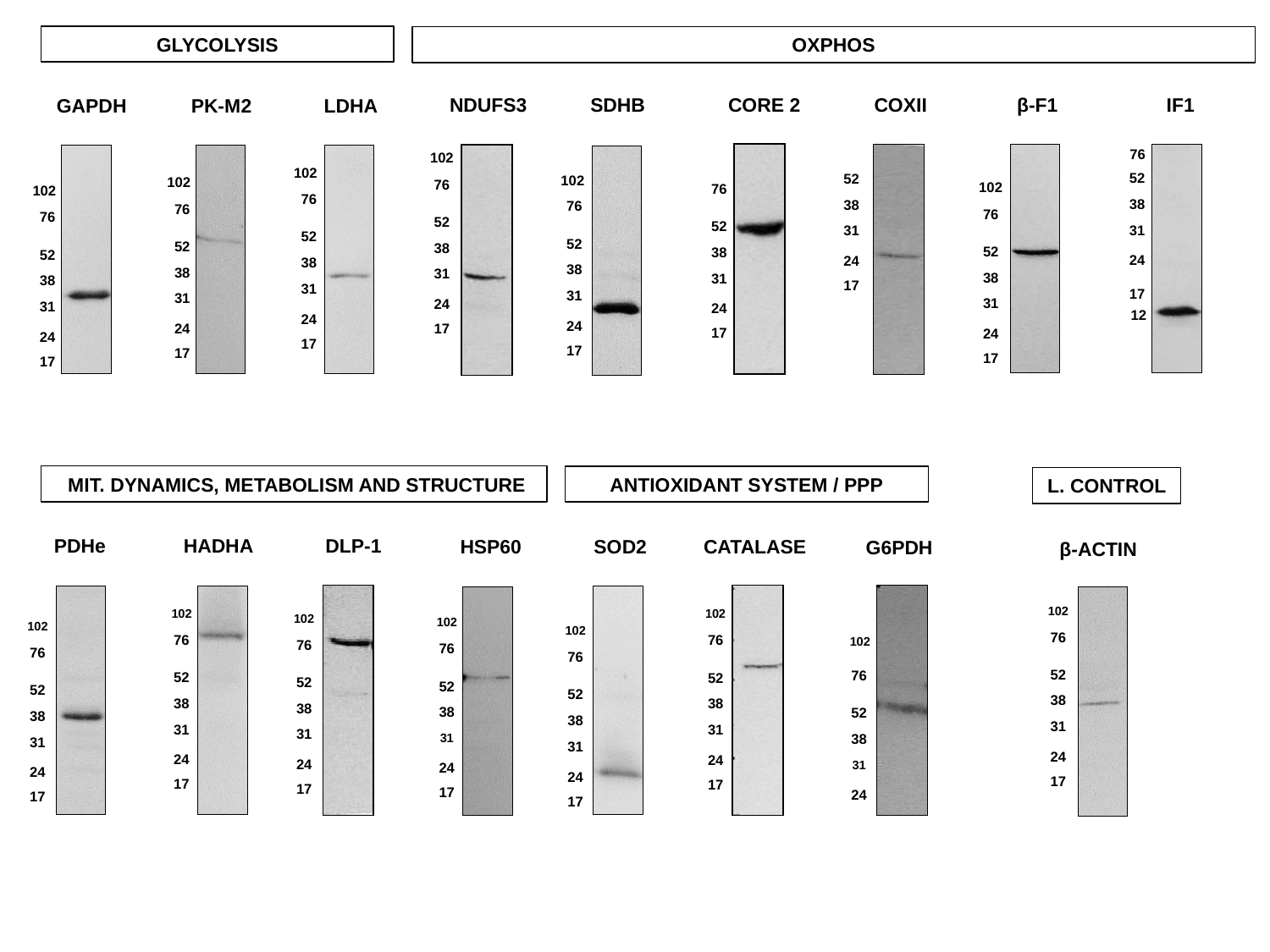

GLYCOLYSIS
OXPHOS
NDUFS3
SDHB
COXII
β-F1
CORE 2
IF1
GAPDH
LDHA
PK-M2
76
102
102
52
52
102
102
76
102
76
102
76
38
38
76
76
76
76
52
52
31
31
52
52
52
38
52
38
52
24
24
38
38
38
31
38
31
38
17
31
17
31
31
31
24
31
24
12
24
24
17
24
17
24
24
17
17
17
17
17
 MIT. DYNAMICS, METABOLISM AND STRUCTURE
ANTIOXIDANT SYSTEM / PPP
L. CONTROL
PDHe
HADHA
DLP-1
CATALASE
SOD2
HSP60
G6PDH
β-ACTIN
102
102
102
102
102
102
102
76
76
76
102
76
76
76
76
52
76
52
52
52
52
52
52
38
38
38
38
38
52
38
38
31
31
31
31
31
38
31
31
24
24
24
24
31
24
24
24
17
17
17
17
17
24
17
17
