# Supplementary figures and images for: Plasma metabolome and skin proteins in Charcot-Marie-Tooth 1A patients
Source: PLoS One. 2017 Jun 2;12(6):e0178376. doi: 10.1371/journal.pone.0178376 (PMC5456076; doi:10.1371/journal.pone.0178376)

## Slide 1
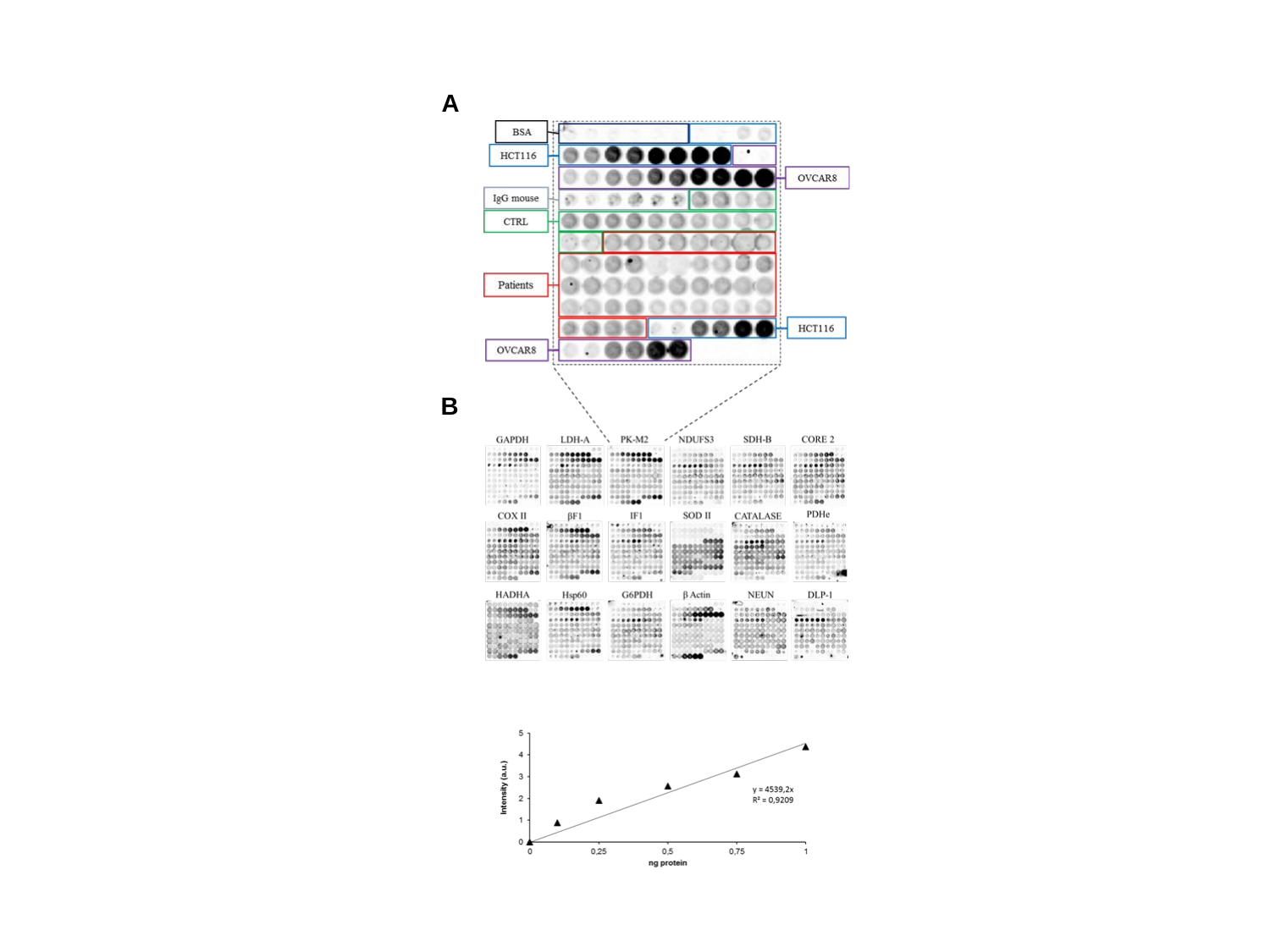

A
B

Supplement: S2 Fig — A, Scheme of RPPmA printing processed for anti-PK-M2 is shown magnified. One nl samples were spotted in duplicate. Black boxed: negative controls of BSA; Blue and purple boxed: standard curves of HCT116 and OVCAR8 cells; Light blue: mouse IgGs; Green boxed: tissue samples from control donors; Red boxed: tissue samples from patients. Below are shown representative RPPmAs processed with other antibodies. B, The plot illustrates the linear correlation that exists between the fluorescence intensity (arbitrary units, a.u.) and the amount of PK-M2 in HCT116 cell lysates. Protein concentrations in the biopsies were calculated according to the fluorescence intensity obtained in the linear plot of HCT116 cells. (PPTX) [file pone.0178376.s005.pptx]
