# Supplementary material for: Plasma metabolome and skin proteins in Charcot-Marie-Tooth 1A patients
Source: PLoS One. 2017 Jun 2;12(6):e0178376. doi: 10.1371/journal.pone.0178376 (PMC5456076; doi:10.1371/journal.pone.0178376)
Supplement: S3 Fig — Extracted ion chromatograms of selected metabolites in plasma samples (purple lines) and commercial standards (blue lines) are shown. The metabolites are defined by their name, ID number, m/z (M+H), retention time, Human Metabolome Data Base ID number and molecular formula. (PPTX) [file pone.0178376.s006.pptx]

## Slide 1
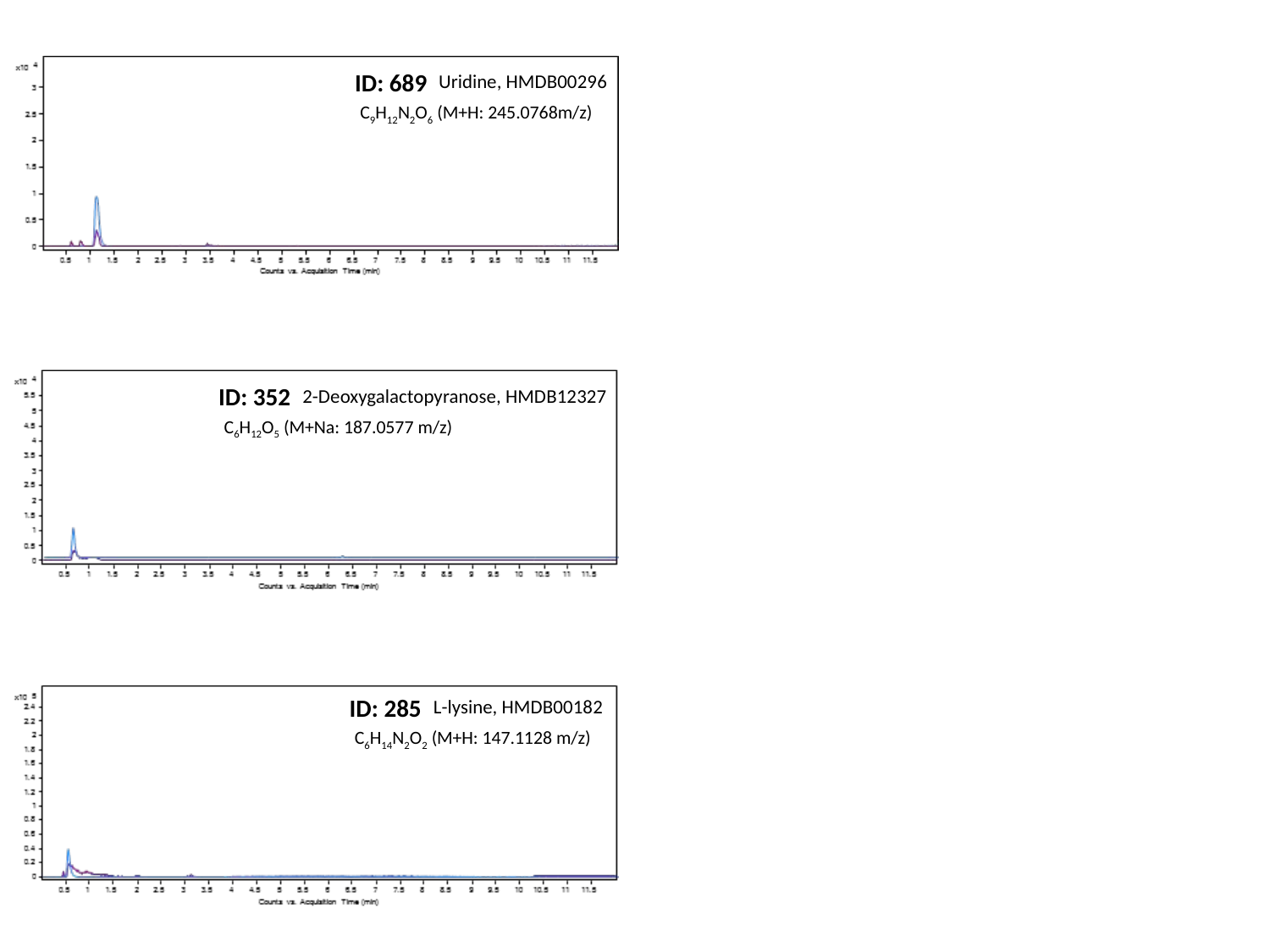

ID: 689
Uridine, HMDB00296
C9H12N2O6 (M+H: 245.0768m/z)
ID: 352
2-Deoxygalactopyranose, HMDB12327
C6H12O5 (M+Na: 187.0577 m/z)
ID: 285
L-lysine, HMDB00182
C6H14N2O2 (M+H: 147.1128 m/z)
